# Characterizing children with chronic kidney disease and severe proteinuria in the United States: a retrospective analysis using electronic health records

**DOI:** 10.1007/s00467-026-07435-y

**Published:** 2026-07-06

**Authors:** Bradley A. Warady, Simone Heeg, Alfredo E. Farjat, Rachel Knapp, Siir Su Saydam, Alexander Vowinkel, Alain Gay, James Potts, Andrea Scalise, Gordana Atanackovic, Sascha van Boemmel-Wegmann

**Affiliations:** 1 Department of Pediatrics, Division of Pediatric Nephrology, University of Missouri-Kansas City School of Medicine, Children’s Mercy Kansas City, Kansas City, MO USA; 2 Real World Data Science & AI (RWDS&AI), Global Medical & Evidence (GME), Bayer AG, Berlin, Germany; 3 Bayer BV, Clinical Statistics & Analytics, Hoofddorp, Netherlands; 4 Real World Evidence Center of Excellence (RWE CoE), Global Medical & Evidence (GME), Bayer AG, Müllerstr. 178, 13353 Berlin, DE Germany; 5 Medical Affairs & Pharmacovigilance (MAPV), Integrated Evidence Generation (IEG), Bayer AG, Berlin, Germany; 6 Studies and Pipeline, Global Medical Affairs (GMA), Bayer AG, Berlin, Germany; 7 Bayer HealthCare Pharmaceuticals Inc., Whippany, NJ USA; 8 Cardiology and Nephrology Clinical Development, Bayer Hispania S.L., San Joan Despi, Spain; 9 Research & Development, Pharmaceutical, Bayer US LLC, Whippany, NJ USA

**Keywords:** Pediatric chronic kidney disease, Proteinuria, Electronic health records, Real-world data

## Abstract

**Background:**

In recent years, real-world data have been increasingly used to enrich data from interventional studies on treatment effectiveness. To explore the viability of hybrid study designs in pediatric chronic kidney disease (CKD), we assessed the feasibility of identifying a real-world cohort of children with CKD and severely increased proteinuria who satisfied adapted eligibility criteria from the FIONA trial.

**Methods:**

We used data from the Optum® de-identified Electronic Health Record dataset (Optum® EHR) to identify and characterize children with CKD and severely increased proteinuria in records from January 2013 to December 2022. Patients were identified based on two sets of estimated glomerular filtration rate (eGFR)/serum creatinine and urine protein-to-creatinine ratio (UPCR) measures within a 90- to 365-day period (final date = index). Patients were required to satisfy adapted eligibility criteria from the FIONA trial and have a baseline prescription for angiotensin-converting enzyme inhibitors (ACEi)/angiotensin receptor blockers (ARBs). Patients were stratified by eGFR and UPCR. Patient characteristics, comorbidities, and medications were descriptively reported.

**Results:**

We identified a total of 381 children with CKD and severely increased proteinuria (median age: 14 years; 52.6% male), among whom 80.8% had eGFR ≥ 60 mL/min/1.73 m^2^ and 69.0% had UPCR > 1 g/g. The patient distribution by CKD stage was: G1, 50.9%; G2, 29.9%; G3–4, 19.2%. Among 397 patients who were excluded given no baseline ACEi/ARB use, > 20% initiated ACEi/ARBs within 6 months following index.

**Conclusions:**

Our study demonstrated the feasibility of identifying a real-world pediatric cohort with CKD and severely increased proteinuria using adapted criteria from the FIONA trial. Findings from this study highlight factors that should be considered in the design of future hybrid studies evaluating treatment effectiveness using data from both trials and the real world.

**Graphical abstract:**

A higher resolution version of the Graphical abstract is available as Supplementary information.

**Figure:**
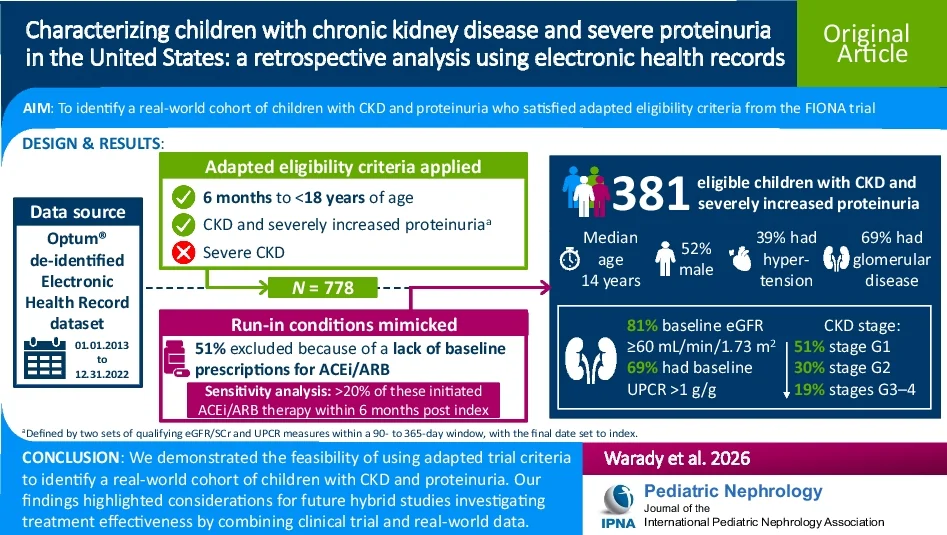

**Supplementary Information:**

The online version contains supplementary material available at https://doi.org/10.1007/s00467-026-07435-y.

## Introduction

Over the past 20 years, the prevalence of pediatric chronic kidney disease (CKD) has been estimated to range from < 0.01% to 1.01%, with the wide variance in estimated rates reflecting issues related to widespread underdiagnosis, given a lack of early symptoms, and routine screening in children [1–3]. As indicated by the *Kidney Disease: Improving Global Outcomes* (KDIGO) guidelines, the assessment and management of children with CKD requires special consideration given the frequent presence of complications and the varying etiologies of CKD in children [4]. Complications of CKD in children include growth impairment that worsens as glomerular filtration rate (GFR) declines [5–7] and anemia, the presence of which is positively correlated with reduced neurocognitive ability and delayed motor development [7–9]. Hypertension, which often manifests in the early stages of CKD in children, increases the risk of cardiovascular (CV) disease and mortality, with the potential to exacerbate adverse outcomes in pediatric patients [10, 11].

Despite this risk, at present, no drugs are indicated for the treatment of CKD in children beyond angiotensin-converting enzyme inhibitors (ACEi) and angiotensin receptor blockers (ARBs), which were first approved over 20 years ago [12]. Instead, current CKD management is based on preventing complications, delaying the progression of CKD, and reducing the risk of kidney failure [4]. ACEi and ARBs are standard-of-care medications in adults with CKD [4]. While they have been shown to slow CKD progression, reduce proteinuria in children [13, 14], and are recommended for use in pediatric CKD with hypertension [15], the evidence base around their optimal use in children is lacking [16]. This absence of novel treatments, along with the profound negative consequences of disease progression on a child’s well-being, highlights the unmet therapeutic need in the area of pediatric CKD.

Finerenone is a selective, non-steroidal, mineralocorticoid receptor antagonist that has been shown to improve CV and kidney outcomes in adult patients with CKD and type 2 diabetes in two large, multicenter, phase 3 randomized clinical trials (FIDELIO-DKD and FIGARO-DKD) [17, 18]. Given its proven efficacy in the adult population, finerenone is now under investigation for use in children with CKD [19]. FIONA is an ongoing randomized, double-blind, placebo-controlled, phase 3 trial evaluating the safety and efficacy of finerenone in addition to ACEi/ARB therapy in children and adolescents (aged 6 months to under 18 years) with CKD [19]. The trial comprises 6 months of treatment, followed by an associated open-label extension study (FIONA OLE) with a treatment period of up to 18 months [19]. The primary outcome is percentage change in urine protein-to-creatinine ratio (UPCR) from baseline to day 180 (± 7), while secondary outcomes include absolute and relative change in estimated GFR (eGFR) and reduction in UPCR by ≥ 30% [20].

Measuring the efficacy and safety of treatment in children is particularly challenging, as stricter regulatory standards apply within the context of pediatric research as compared with interventional trials in adults. Obtaining adequate sample sizes presents a common challenge, as some conditions are particularly rare in children, and ethical considerations further limit both patient enrollment and outcome selection [21]. Electronic health records (EHRs) provide information on the real-world profile and treatment of patients in clinical practice. Combined with their broader generalizability and long-term follow-up, they offer a unique opportunity for enrichment of clinical trial data on disease progression and treatment efficacy with outcomes observed in real-world clinical practice [22, 23]. Nevertheless, ensuring comparability of cohorts and clinically relevant endpoints is of utmost importance when evaluating evidence generated across clinical trials and real-world data (RWD). To ensure appropriate comparisons, sufficiently large sample sizes as well as effectively translated eligibility criteria and outcome definitions are required, offering an appropriate basis for adjustment using commonly applied matching or weighting methods.

To explore the viability of future hybrid study designs in pediatric CKD leveraging data from both RWD and the FIONA trial, our study aimed to assess the feasibility of using EHR data from the United States to identify and characterize a real-world pediatric population with CKD and severely increased proteinuria [19, 24]. In addition to identifying a base cohort of patients satisfying adapted eligibility criteria from the FIONA trial, stratified by eGFR and UPCR at baseline, this study sought to describe their sociodemographic, clinical, and treatment characteristics.

## Methods

### Study design and data sources

This was a retrospective, observational cohort study that included pediatric patients with CKD and severely increased proteinuria who satisfied adapted criteria from the FIONA trial using patient-level data from the Optum® de-identified Electronic Health Record dataset (Optum® EHR) covering the period from January 1, 2013, to December 31, 2022 (Supplementary Fig. 1, Supplementary Fig. 2). Optum® EHR is a longitudinal electronic health record repository derived from dozens of health care provider organizations in the US across the continuum of care. Population-level data utilized for the purposes of this study included sociodemographic characteristics, laboratory results, and comedication prescriptions as well as diagnostic and procedure codes [24] (Supplementary Table 1, Supplementary Table 2).

### Patient population

**Fig. 1:**
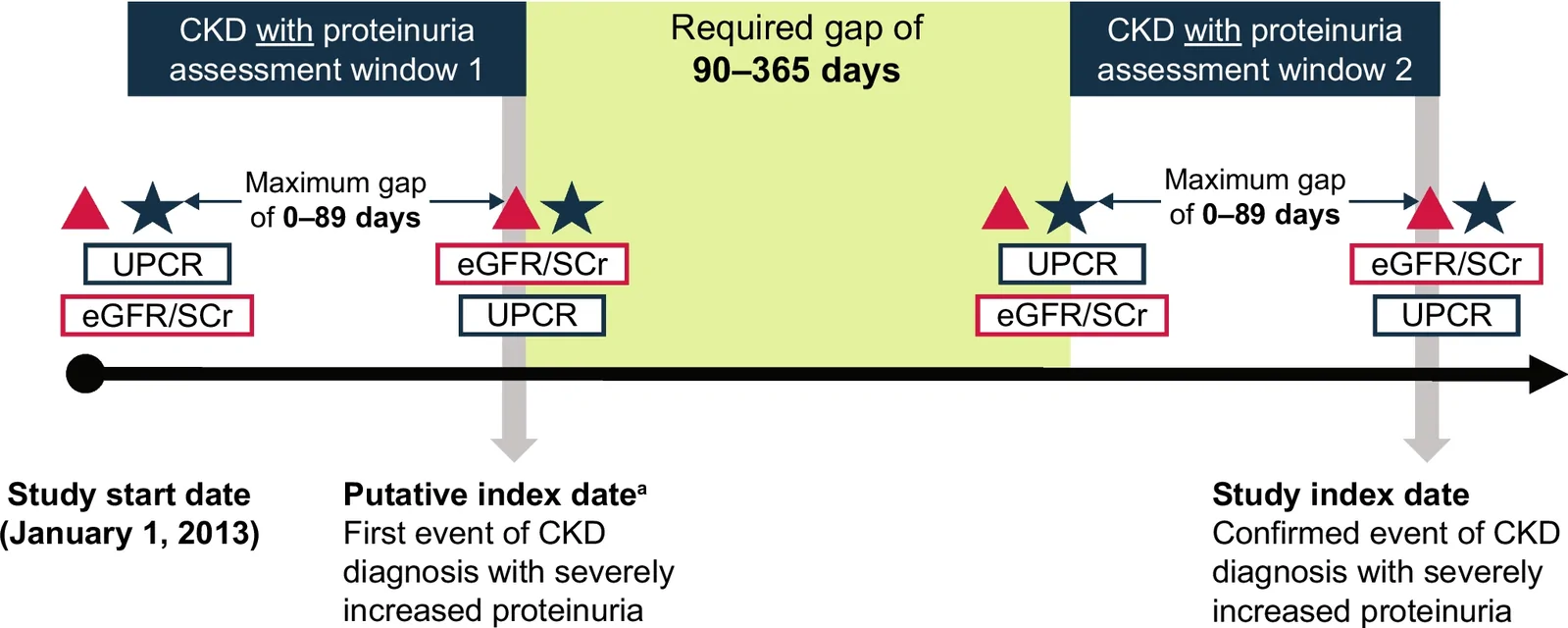
Index date by laboratory confirmation of CKD with severely increased proteinuria. ^a^The putative index date will be assigned to the latest occurring lab measure of either UPCR or eGFR/SCr. To be included in the cohort, CKD diagnosis criteria had to be satisfied ≥ 2 times occurring 90–365 days apart, with the latest putative index date being assigned as the study index date. *CKD* chronic kidney disease, *eGFR* estimated glomerular filtration rate, *SCr* serum creatinine, *UPCR* urine protein-to-creatinine ratio

Pediatric patients were identified for inclusion in the study based on the presence of two pairs of qualifying laboratory results, separated by a period of 90 to 365 days. Each pair consisted of one eGFR or serum creatinine (SCr) measurement and one UPCR measurement indicating severely increased proteinuria (see Table 1 for age-specific eGFR and UPCR criteria), with the two tests taken ≤ 89 days apart, as illustrated in Fig. 1. The index date for the study was defined as the date of the last (fourth) qualifying event (either SCr, eGFR, or UPCR), indicating CKD with severely increased proteinuria.

**Table 1:** CKD and UPCR eligibility criteria by age group

Age	CKD stage	UPCR^a^
2 to < 18 years	**CKD stage G2:** eGFR 60 to < 90 mL/min/1.73 m^2^	≥ 0.5 g/g
	**CKD stage G3:** eGFR 30 to < 60 mL/min/1.73 m^2^	
	**CKD stage G1:** eGFR ≥ 90 mL/min/1.73 m^2^	≥ 1.0 g/g
1 to < 2 years	**CKD stage G1:** eGFR ≥ 90 mL/min/1.73 m^2^	≥ 1.0 g/g
	**CKD stage G2:** eGFR 60 to < 90 mL/min/1.73 m^2^	
	**CKD stage G3:** eGFR 30 to < 60 mL/min/1.73 m^2^	
6 months to < 1 year	**SCr** ≤ 0.4 mg/dL	≥ 1.0 g/g

^a^Threshold for severely increased proteinuria

*CKD* chronic kidney disease, *eGFR* estimated glomerular filtration rate, *G* GFR-based, *SCr* serum creatinine, *UPCR* urine protein-to-creatinine ratio

For cohort creation, eGFR was calculated using serum creatinine values in association with data on height and age, using the following Chronic Kidney Disease in Children Under 25 (CKiD U25) formula [25]:

$$eGFR=K \times height \left(cm\right)/ SCr$$

where *K* was based on age and sex. UPCR was evaluated using median daily values derived from urine samples.

Following the initial case extraction step, further eligibility criteria were applied. Patients were required to be 6 months to < 18 years of age at index, and to have at least 180 days of baseline (pre-index) data (Supplementary Table 3). Subsequently, exclusion conditions were applied in line with the criteria outlined in the FIONA trial. Patients were excluded if they had severe CKD based on two or more corresponding eGFR/SCr measurements taken at least 90 days apart (eGFR < 30 mL/min/1.73 m^2^ in patients 1 to < 18 years of age; SCr > 0.4 mg/dL in patients < 1 year of age). Patients were also excluded if they had previously received a kidney transplant or had a prior record of unstable kidney function, high serum potassium levels, other kidney diseases and liver disease (a complete list of adapted eligibility criteria is included in Supplementary Table 3).

In a final step, we excluded patients without a baseline prescription for ACEi/ARB from the base cohort (Supplementary Fig. 2). This was done to mimic the conditions of the FIONA trial, which includes a 90-day run-in phase prior to screening, during which patients are intended to be initiated, optimized, and stabilized on ACEi/ARB [19].

The base cohort was stratified by index eGFR (≥ 60 mL/min/1.73 m^2^ vs. < 60 mL/min/1.73 m^2^) and UPCR (≤ 1 g/g or > 1 g/g). Finally, we included a sensitivity cohort comprising patients without baseline ACEi/ARB prescriptions that were excluded at the last attrition step.

### Study variables

In addition to sample size, we reported the following outcomes at index and/or during the 180-day baseline period for the base cohort, subgroups, and sensitivity cohort: sociodemographic characteristics (e.g., age, sex, and race), clinical characteristics (e.g., eGFR, UPCR, serum potassium, CKD stage, CKD etiology), comorbidities, and baseline medication use. If the sample size for a particular characteristic was nonzero but smaller than five patients, n was reported as < 5. Finally, given the large number of patients excluded during the last attrition step, a post hoc sensitivity analysis was performed to evaluate time to initiation of ACEi/ARB in the sensitivity cohort.

### Statistical analysis

For descriptive analyses, categorical variables were reported using frequency tables (counts and percentages), while continuous variables were reported using summary statistics including mean and standard deviation or median and interquartile (Q1, Q3) ranges (IQRs), depending on the skewness of the distribution. Unreported variables were reported as “Unknown” in descriptive tables.

In addition to these descriptive analyses, the sensitivity analysis consisted of time to event analyses using Aalen–Johansen estimates to account for the competing risk of death; cumulative incidence of ACEi/ARB initiation in treatment-naïve patients was reported quarterly over 3 years. Incidence rates of ACEi/ARB initiation per 100 patient-years were also reported.

Data were extracted and cleaned by removing null and invalid values and standardizing units using Python (Version 3.9). Statistical analyses were conducted in R (Version 4.3.2).

## Results

### Identification of study population

A total of 1136 patients with pediatric CKD and severely increased proteinuria were identified in Optum® EHR based on eGFR/SCr and UPCR criteria. Of these, 778 satisfied the eligibility criteria adapted from the FIONA trial. However, 397 (51.0%) had no baseline prescriptions for ACEi/ARB and therefore did not match the profile of pediatric patients included in the FIONA trial population (after run-in). The proportion of patients excluded based on a lack of ACEi/ARB prescriptions (sensitivity cohort) was highest in the 2 to < 6 years age group with 59 patients (69.4%), followed by the 6 to < 12 years age group with 127 (58.0%), and the 12 to < 18 years age group with 210 (44.4%). All patients (100.0%) under 2 years of age (< 5) were also excluded from the base cohort due to a lack of baseline ACEi/ARB treatment.

### Base cohort

#### Sociodemographic characteristics

The final base cohort included 381 patients with pediatric CKD and severely increased proteinuria who satisfied all adapted trial criteria (Supplementary Fig. 2). The median age of patients was 14.0 years (IQR 10, 16). All children were ≥ 2 years of age, and there were slightly more males than females (52.0% vs. 48.0%), as shown in Table 2.

**Table 2:** Sociodemographic characteristics in the overall base cohort and in baseline eGFR and UPCR subgroups

Characteristic	Base cohort (overall) (*N* = 381)	Baseline eGFR		Baseline UPCR	
		≥ 60 mL/min/1.73 m^2^ (*n* = 308)	< 60 mL/min/1.73 m^2^ (*n* = 73)	≤ 1 g/g (*n* = 118)	> 1 g/g (*n* = 263)
Age in years, median (Q1, Q3)	14.0 (10.0, 16.0)	14.0 (10.0, 16.0)	13.0 (11.0, 16.0)	13.0 (10.0, 15.0)	14.0 (11.0, 16.0)
Age group, *n* (%)					
≥ 6 months to < 2 years	0	0	0	0	0
≥ 2 to < 6 years	26 (6.8)	24 (7.8)	< 5 (< 6.8)	8 (6.8)	18 (6.8)
≥ 6 to < 12 years	92 (24.1)	72 (23.4)	20 (27.4)	34 (28.8)	58 (22.1)
≥ 12 to < 18 years	263 (69.0)	212 (68.8)	51 (69.9)	76 (64.4)	187 (71.1)
Sex, *n* (%)					
Female	183 (48.0)	152 (49.4)	31 (42.5)	57 (48.3)	126 (47.9)
Male	198 (52.0)	156 (50.6)	42 (57.5)	61 (51.7)	137 (52.1)
Race, *n* (%)					
African American	61 (16.0)	50 (16.2)	11 (15.1)	18 (15.3)	43 (16.3)
Asian	24 (6.3)	21 (6.8)	< 5 (< 6.8)	7 (5.9)	17 (6.5)
White	218 (57.2)	170 (55.2)	48 (65.8)	68 (57.6)	150 (57.0)
Other/unknown	78 (20.5)	67 (21.8)	11 (15.1)	25 (21.2)	53 (20.2)

*eGFR* estimated glomerular filtration rate, *Q* quartile, *UPCR* urine protein-to-creatinine ratio

#### Clinical characteristics

Mean ± standard deviation eGFR at index was 93.5 ± 36.3 mL/min/1.73 m^2^, while median UPCR was 1.5 g/g (IQR 0.9, 3.0). Overall, 194 patients (50.9%) had CKD stage G1, 114 (29.9%) had stage G2, 72 (18.9%) had stage G3a/b, and < 5 (< 1.3%) had stage G4. Glomerular disease was reported as the CKD etiology for 262 patients (68.8%), whereas 37 (9.7%) had non-glomerular disease, and 82 (21.5%) had an unknown CKD etiology. Focal segmental glomerulosclerosis accounted for 32 (8.4%) of the individual diagnoses, followed by 23 diagnoses (6.0%) of immunoglobulin A nephropathy, and 17 (4.5%) of Alport syndrome (Table 3). The most common comorbidities were hypertension, which was reported in 150 (39.4%) patients, followed by anemia in 52 (13.7%), urinary tract infection in 34 (8.9%), hyperlipidemia in 30 (7.9%), and growth retardation in 27 (7.1%), as shown in Table 3.

**Table 3:** Clinical characteristics in the overall base cohort and in baseline eGFR and UPCR subgroups

Characteristic	Base cohort (overall) (*N* = 381)	Baseline eGFR		Baseline UPCR	
		≥ 60 mL/min/1.73 m^2^ (*n* = 308)	< 60 mL/min/1.73 m^2^ (*n* = 73)	≤ 1 g/g (*n* = 118)	> 1 g/g (*n* = 263)
Premature birth, *n* (%)	< 5 (< 1.3)	< 5 (< 1.6)	< 5 (< 6.8)	0	< 5 (< 1.9)
Comorbidities, *n* (%)					
Type 2 diabetes	15 (3.9)	12 (3.9)	< 5 (< 6.8)	< 5 (< 4.2)	12 (4.6)
Heart failure	< 5 (< 1.3)	< 5 (< 1.6)	0	0	1 (0.4)
Urinary tract infection	34 (8.9)	23 (7.5)	11 (15.1)	14 (11.9)	20 (7.6)
Hypertension	150 (39.4)	112 (36.4)	38 (52.1)	47 (39.8)	103 (39.2)
Growth retardation	27 (7.1)	19 (6.2)	8 (11.0)	12 (10.2)	15 (5.7)
Anemia	52 (13.6)	34 (11.0)	18 (24.7)	18 (15.3)	34 (12.9)
Hyperlipidemia	30 (7.9)	20 (6.5)	10 (13.7)	8 (6.8)	22 (8.4)
Metabolic bone disease	< 5 (< 1.3)	< 5 (< 1.6)	< 5 (< 6.8)	0	< 5 (< 1.9)
Cardiomyopathy	< 5 (< 1.3)	< 5 (< 1.6)	0	0	< 5 (< 1.9)
Congenital heart malformation	9 (2.4)	< 5 (< 1.6)	6 (8.2)	< 5 (< 4.2)	5 (1.9)
eGFR, mL/min/1.73 m^2^, mean ± SD	93.5 ± 36.3	104.9 ± 30.6	45.2 ± 8.2	78.0 ± 26.3	100.4 ± 38.1
eGFR, *n* (%)					
< 30 mL/min/1.73 m^2^	< 5 (< 1.3)	0	< 5 (< 6.8)	< 5 (< 4.2)	0
≥ 30 to < 60 mL/min/1.73 m^2^	72 (18.9)	0	72 (98.6)	31 (26.3)	41 (15.6)
≥ 60 mL/min/1.73 m^2^	308 (80.8)	308 (100.0)	0	86 (72.9)	222 (84.4)
Log(UPCR), g/g, mean ± SD	0.5 ± 0.9	0.5 ± 0.9	0.3 ± 0.8	−0.5 ± 0.5	0.9 ± 0.7
UPCR, *n* (%)					
< 0.5 g/g^a^	22 (5.8)	19 (6.2)	< 5 (< 6.8)	22 (18.6)	0
≥ 0.5 to ≤ 1.0	96 (25.2)	67 (21.8)	29 (39.7)	96 (81.4)	0
> 1.0 g/g	263 (69.0)	222 (72.1)	41 (56.2)	0	263 (100.0)
Serum potassium, *n* (%)					
> 4.5 mmol/L	81 (21.3)	50 (16.2)	31 (42.5)	27 (22.9)	54 (20.5)
≤ 4.5 mmol/L	300 (78.7)	258 (83.8)	42 (57.5)	91 (77.1)	209 (79.5)
CKD stage, *n* (%)					
G1	194 (50.9)	194 (63.0)	0	36 (30.5)	158 (60.1)
G2	114 (29.9)	114 (37.0)	0	50 (42.4)	64 (24.3)
G3a	41 (10.8)	0	41 (56.2)	19 (16.1)	22 (8.4)
G3b	31 (8.1)	0	31 (42.5)	12 (10.2)	19 (7.2)
G4	< 5 (< 1.3)	0	< 5 (< 6.8)	< 5 (< 4.2)	0
CKD etiology, glomerular vs. non-glomerular, *n* (%)					
Glomerular disease	262 (68.8)	227 (73.7)	35 (47.9)	74 (62.7)	188 (71.5)
Non-glomerular disease	37 (9.7)	13 (4.2)	24 (32.9)	20 (16.9)	17 (6.5)
Unknown	82 (21.5)	68 (22.1)	14 (19.2)	24 (20.3)	58 (22.1)
CKD etiology with ≥ 1 diagnostic code, *n* (%)					
Alport syndrome	17 (4.5)	15 (4.9)	< 5 (< 6.8)	5 (4.2)	12 (4.6)
Diabetic nephropathy	6 (1.6)	5 (1.6)	< 5 (< 6.8)	< 5 (< 4.2)	5 (1.9)
Focal segmental glomerulosclerosis	32 (8.4)	27 (8.8)	5 (6.8)	< 5 (< 4.2)	28 (10.6)
IgA nephropathy	23 (6.0)	22 (7.1)	< 5 (< 6.8)	9 (7.6)	14 (5.3)
Other glomerular diseases	248 (65.1)	212 (68.8)	36 (49.3)	68 (57.6)	180 (68.4)
Non-glomerular congenital anomalies	31 (8.1)	9 (2.9)	22 (30.1)	20 (16.9)	11 (4.2)
Other non-glomerular diseases	56 (14.7)	39 (12.7)	17 (23.3)	19 (16.1)	37 (14.1)
Any glomerular disease^b^	262 (68.8)	227 (73.7)	35 (47.9)	74 (62.7)	188 (71.5)
Any non-glomerular disease^c^	62 (16.3)	32 (10.4)	30 (41.1)	27 (22.9)	35 (13.3)

^a^Although UPCR ≥ 0.5 g/g was required for enrollment, patients were not disqualified if they had UPCR < 0.5 g/g at some point post initial CKD assessment

^b^Glomerular disease defined as ≥ 2 diagnostic codes or 1 diagnostic code and 1 biopsy code

^c^Any non-glomerular disease with ≥ 2 diagnostic codes

*CKD* chronic kidney disease, *eGFR* estimated glomerular filtration rate, *G* GFR-based, *IgA* immunoglobulin A, *SD* standard deviation, *UPCR* urine protein-to-creatinine ratio

#### Baseline comedications

In addition to ACEi/ARBs, 196 patients (51.4%) in the base cohort were prescribed corticosteroids, and 177 (46.5%) received disease-modifying antirheumatic drugs and immunosuppressive medications at baseline (Table 4). No patients had prescriptions for diabetes drugs, renin inhibitors, or certain classes of diuretics (carbonic anhydrase inhibitors, vasopressin antagonists, classes other than those specified in Table 4).

**Table 4:** Baseline comedications in the overall base cohort and in baseline eGFR and UPCR subgroups

Prescription drug,^a^ *n* (%)	Base cohort (overall) (*N* = 381)	Baseline eGFR		Baseline UPCR	
		≥ 60 mL/min/1.73 m^2^ (*n* = 308)	< 60 mL/min/1.73 m^2^ (*n* = 73)	≤ 1 g/g (*n* = 118)	> 1 g/g (*n* = 263)
Antiarrhythmics	22 (5.8)	13 (4.2)	9 (12.3)	4 (3.4)	18 (6.8)
Antihypertensives	381 (100.0)	308 (100.0)	73 (100.0)	118 (100.0)	263 (100.0)
Adrenergic receptor agonists	14 (3.7)	9 (2.9)	5 (6.8)	< 5 (< 4.2)	10 (3.8)
ARBs	66 (17.3)	52 (16.9)	14 (19.2)	16 (13.6)	50 (19.0)
ACE inhibitors	350 (91.9)	285 (92.5)	65 (89.0)	108 (91.5)	242 (92.0)
CCBs	49 (12.9)	35 (11.4)	14 (19.2)	14 (11.9)	35 (13.3)
Other antihypertensives	< 5 (< 1.3)	< 5 (< 1.6)	0	< 5 (< 4.2)	< 5 (< 1.9)
Diuretics	41 (10.8)	34 (11.0)	7 (9.6)	6 (5.1)	35 (13.3)
Loop or high-ceiling diuretics	29 (7.6)	25 (8.1)	< 5 (< 6.8)	< 5 (< 4.2)	25 (9.5)
Osmotic diuretics	< 5 (< 1.3)	< 5 (< 1.6)	0	0	< 5 (< 1.9)
Potassium-sparing diuretics	< 5 (< 1.3)	< 5 (< 1.6)	0	< 5 (< 4.2)	< 5 (< 1.9)
Thiazides or low-ceiling diuretics	11 (2.9)	8 (2.6)	< 5 (< 6.8)	0	11 (4.2)
Blood and related drugs	7 (1.8)	< 5 (< 1.6)	6 (8.2)	< 5 (< 4.2)	6 (2.3)
Erythropoiesis-stimulating drugs	7 (1.8)	< 5 (< 1.6)	6 (8.2)	< 5 (< 4.2)	6 (2.3)
Anti-infectives	105 (27.6)	81 (26.3)	24 (32.9)	31 (26.3)	74 (28.1)
Antibacterials	105 (27.6)	81 (26.3)	24 (32.9)	31 (26.3)	74 (28.1)
Corticosteroids	196 (51.4)	170 (55.2)	26 (35.6)	64 (54.2)	132 (50.2)
DMARDs and immunosuppressive medications without corticosteroids	177 (46.5)	160 (51.9)	17 (23.3)	46 (39.0)	131 (49.8)
Antineoplastic immunosuppressive medications	< 5 (< 1.3)	< 5 (< 1.6)	0	< 5 (< 4.2)	< 5 (< 1.9)
Biologic DMARDs^b^	< 5 (< 1.3)	< 5 (< 1.6)	0	0	< 5 (< 1.9)
Non-biologic DMARDs^c^	166 (43.6)	152 (49.4)	14 (19.2)	43 (36.4)	123 (46.8)

Drug classes with zero counts were not included in the table

^a^Patients could have recorded use of more than one drug under each category

^b^Such as adalimumab, eculizumab, ustekinumab, or etanercept

^c^Such as mycophenolate mofetil, tacrolimus, or hydroxychloroquine sulfate

*ACE* angiotensin-converting enzyme, *ARB* angiotensin receptor blocker, *CCB* calcium channel blocker, *DMARD* disease-modifying antirheumatic drug, *eGFR* estimated glomerular filtration rate, *UPCR* urine protein-to-creatinine ratio

#### eGFR subgroups

Among 381 patients, 308 (80.8%) had a baseline eGFR ≥ 60 mL/min/1.73 m^2^, and 73 (19.2%) had a baseline eGFR < 60 mL/min/1.73 m^2^. Male and White patients accounted for a higher share of patients in the lower eGFR group (Male: 57.5%; White: 65.8%) compared with the eGFR ≥ 60 mL/min/1.73 m^2^ subgroup (Male: 50.6%; White: 55.2%, Table 2). Hypertension, anemia, urinary tract infections, hyperlipidemia, and congenital heart malformation were observed more often in patients with an eGFR < 60 mL/min/1.73 m^2^ (Table 3). Baseline use of corticosteroids, and disease-modifying antirheumatic drugs and immunosuppressive medications was higher in patients with an eGFR ≥ 60 mL/min/1.73 m^2^, while the use of antiarrhythmics and erythropoiesis-stimulating agents was higher in those with an eGFR < 60 mL/min/1.73 m^2^ (Table 4).

#### UPCR subgroup

Among a total of 381 patients, 118 (31.0%) had a baseline UPCR ≤ 1 g/g, and 263 (69.0%) had a baseline UPCR > 1 g/g. Sociodemographic characteristics were generally similar between the two UPCR subgroups (Table 2). In the UPCR > 1 g/g subgroup, 158 patients (60.1%) had CKD stage G1, with progressively fewer patients experiencing more advanced stages (stage G2 24.3%; stage G3a/b 15.6%, stage G4 0). The distribution of patients in the UPCR ≤ 1 g/g subgroup was skewed toward more advanced stages, with 81 (68.6%) categorized as stages G2–G3b, and < 5 (< 4.2%) patients with CKD stage G4 (Table 3). A higher proportion of patients in the UPCR > 1 g/g subgroup were treated with disease-modifying antirheumatic drugs and immunosuppressive medications at baseline than in the UPCR ≤ 1 g/g subgroup (Table 4).

### Sensitivity analysis

#### Characteristics of the sensitivity cohort

The 397 patients in the sensitivity cohort (Table 5, Supplementary Fig. 2) appeared to be younger on average than those in the base cohort, with a median age of 12 years (IQR 8.0, 15.0) compared with 14 (IQR 10.0, 16.0) years in the base cohort. There were similar proportions of males (sensitivity cohort: 52.6%; base cohort: 52.0%) and White patients (sensitivity cohort: 58.7%; base cohort: 57.2%) in both cohorts. In terms of clinical characteristics, the distribution of patients across CKD stages and eGFR, UPCR, and serum potassium categories was similar in both cohorts (Table 5). Although no hypothesis testing was performed within the context of this primarily descriptive study, the sensitivity cohort had a lower reported prevalence of hypertension (15.9% vs. 39.4% in the base cohort), and a lower level of prescriptions for any antihypertensive medication, including ACEi/ARBs, adrenergic receptor agonists, calcium channel blockers, and other antihypertensives (8.1% vs. 100.0%, respectively; Table 5).

**Table 5:** Baseline characteristics of the base and sensitivity cohorts

Characteristic	Base cohort (overall) (*N* = 381)	Sensitivity cohort (without BL ACEi/ARB) (*N* = 397)
*Demographics*		
Age in years, median (Q1, Q3)	14.0 (10.0, 16.0)	12.0 (8.0, 15.0)
Age group, *n* (%)		
≥ 6 months to < 2 years	0	< 5 (< 1.3)
≥ 2 to < 6 years	26 (6.8)	59 (14.9)
≥ 6 to < 12 years	92 (24.1)	127 (32.0)
≥ 12 to < 18 years	263 (69.0)	210 (52.9)
Sex, *n* (%)		
Female	183 (48.0)	188 (47.4)
Male	198 (52.0)	209 (52.6)
Race, *n* (%)		
African American	61 (16.0)	62 (15.6)
Asian	24 (6.3)	38 (9.6)
White	218 (57.2)	233 (58.7)
Other/Unknown	78 (20.5)	64 (16.1)
*Clinical characteristics*		
Comorbidities, *n* (%)		
Type 2 diabetes	15 (3.9)	7 (1.8)
Heart failure	< 5 (< 1.3)	0
Urinary tract infection	34 (8.9)	29 (7.3)
Hypertension	150 (39.4)	63 (15.9)
Growth retardation	27 (7.1)	28 (7.1)
Anemia	52 (13.6)	40 (10.1)
Hyperlipidemia	30 (7.9)	10 (2.5)
Metabolic bone disease	< 5 (< 1.3)	7 (1.8)
Cardiomyopathy	< 5 (< 1.3)	0
Congenital heart malformation	9 (2.4)	10 (2.5)
eGFR, mL/min/1.73 m^2^, mean ± SD	93.5 ± 36.3	101.5 ± 50.4
eGFR, *n* (%)		
< 30 mL/min/1.73 m^2^	< 5 (< 1.3)	0
≥ 30 to < 60 mL/min/1.73 m^2^	72 (18.9)	71 (17.9)
≥ 60 mL/min/1.73 m^2^	308 (80.8)	326 (82.1)
Log(UPCR), g/g, mean ± SD	0.5 ± 0.9	0.6 ± 1.0
UPCR, *n* (%)		
< 0.5 g/g^a^	22 (5.8)	23 (5.8)
≥ 0.5 to ≤ 1.0	96 (25.2)	88 (22.2)
> 1.0 g/g	263 (69.0)	286 (72.0)
Serum potassium, *n* (%)		
> 4.5 mmol/L	81 (21.3)	73 (18.4)
≤ 4.5 mmol/L	300 (78.7)	317 (79.9)
CKD stage, *n* (%)		
G1	194 (50.9)	241 (60.7)
G2	114 (29.9)	85 (21.4)
G3a	41 (10.8)	37 (9.3)
G3b	31 (8.1)	34 (8.6)
G4	< 5 (< 1.3)	0
CKD etiology, glomerular vs. non‑glomerular, *n* (%)		
Glomerular disease	262 (68.8)	229 (57.7)
Non-glomerular disease	37 (9.7)	62 (15.6)
Unknown	82 (21.5)	106 (26.7)
CKD etiology with ≥ 1 diagnostic code, *n* (%)		
Alport syndrome	17 (4.5)	4 (1.0)
Diabetic nephropathy	6 (1.6)	0
Focal segmental glomerulosclerosis	32 (8.4)	15 (3.8)
IgA nephropathy	23 (6.0)	22 (5.5)
Other glomerular diseases	248 (65.1)	234 (58.9)
Non-glomerular congenital anomalies	31 (8.1)	40 (10.1)
Other non-glomerular diseases	56 (14.7)	53 (13.4)
Any glomerular diseases^b^	262 (68.8)	229 (57.7)
Any non-glomerular diseases^c^	62 (16.3)	62 (15.6)
*Comedications*		
Antiarrhythmics, *n* (%)	22 (5.8)	11 (2.8)
Antiarrhythmics, class II	22 (5.8)	11 (2.8)
CCB with antiarrhythmic effects	0	0
Antihypertensives, *n* (%)	381 (100.0)	32 (8.1)
Adrenergic receptor agonists	14 (3.7)	8 (2.0)
ARBs	66 (17.3)	0
ACE inhibitors	350 (91.9)	0
CCBs	49 (12.9)	24 (6.1)
Renin inhibitors	0	0
Other antihypertensives	< 5 (< 1.3)	< 5 (< 1.3)
Diuretics, *n* (%)	41 (10.8)	45 (11.3)
Carbonic anhydrase inhibitors	0	< 5 (< 1.3)
Loop or high-ceiling diuretics	29 (7.6)	36 (9.1)
Osmotic diuretics	< 5 (< 1.3)	0
Potassium-sparing diuretics	< 5 (< 1.3)	< 5 (< 1.3)
Thiazides or low-ceiling diuretics	11 (2.9)	12 (3.0)
Vasopressin antagonists	0	0
Other diuretics	0	0
Blood and related drugs, *n* (%)	7 (1.8)	< 5 (< 1.3)
Erythropoiesis-stimulating drugs	7 (1.8)	< 5 (< 1.3)
Anti-infectives, *n* (%)	105 (27.6)	78 (19.7)
Antibacterials	105 (27.6)	78 (19.7)
Corticosteroids, *n* (%)	196 (51.4)	184 (46.4)
DMARDs and immunosuppressive medications without corticosteroids, *n* (%)	177 (46.5)	126 (31.7)
Antineoplastic immunosuppressive medications	< 5 (< 1.3)	< 5 (< 1.3)
Biologic DMARDs^d^	< 5 (< 1.3)	< 5 (< 1.3)
Non-biologic DMARDs^e^	166 (43.6)	103 (25.9)

^a^Although UPCR ≥ 0.5 g/g was required for enrollment, patients were not disqualified if they had UPCR < 0.5 g/g at some point following the initial CKD assessment

^b^Glomerular disease defined as two or more diagnostic codes or one diagnostic code and one biopsy code

^c^Any non-glomerular disease with two or more diagnostic codes

^d^Such as adalimumab, eculizumab, ustekinumab, and etanercept

^e^Such as mycophenolate mofetil, tacrolimus, and hydroxychloroquine sulfate

*ACE* angiotensin-converting enzyme, *ARB* angiotensin receptor blocker, *BL* baseline, *CCB* calcium channel blocker, *CKD* chronic kidney disease, *DMARD* disease-modifying antirheumatic drug, *eGFR* estimated glomerular filtration rate, *G* GFR-based, *IgA* immunoglobulin A, *Q* quartile, *SD* standard deviation, *UPCR* urine protein-to-creatinine ratio

#### Treatment initiation with ACEi/ARB

**Fig. 2:**
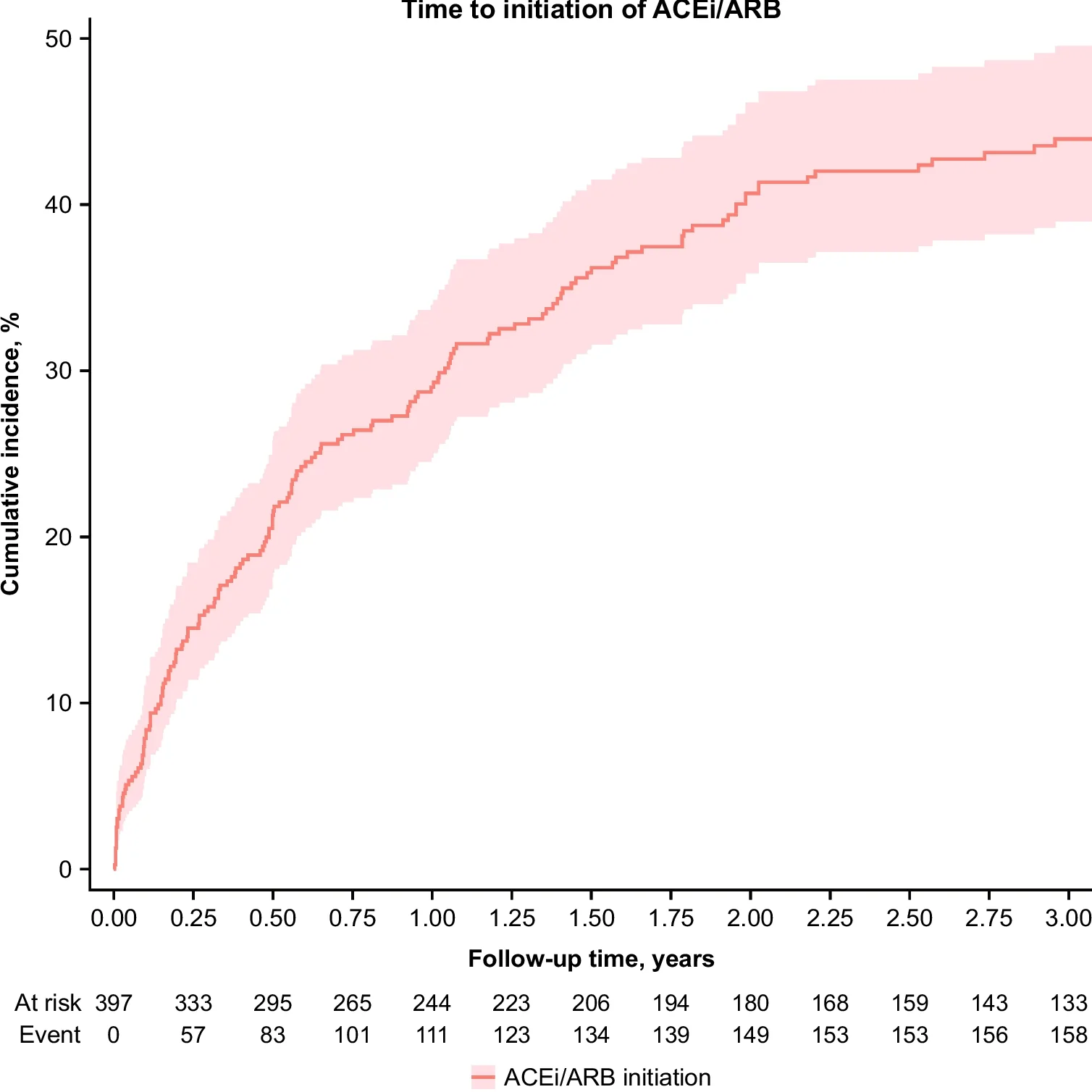
Sensitivity analysis on time to initiation of ACEi/ARB treatment in the sensitivity cohort (*N* = 397). Cumulative event incidence based on Aalen–Johansen estimates with censorship at first ACEi/ARB initiation event (or competing risk of all-cause mortality). *ACEi* angiotensin-converting enzyme inhibitor, *ARB* angiotensin receptor blocker

The cumulative incidence of ACEi/ARB initiation in the sensitivity cohort is shown in Fig. 2. Within the first 6 months of follow-up, the incidence rate of ACEi/ARB initiation was 49.4 events per 100 patient-years, and over 20% of these treatment-naïve patients were prescribed an ACEi/ARB.

## Discussion

This real-world, retrospective observational study assessed the feasibility of identifying a pediatric cohort of patients with CKD and severely increased proteinuria using Optum® EHR in alignment with eligibility criteria adapted from the FIONA trial. Overall, 381 patients were identified for inclusion in the base study cohort. Among these patients, none were < 2 years of age and > 90% were 6 years of age or older. Most patients in the base cohort had early-stage CKD, and disease etiology was glomerular in > 65% of patients. Over a third of the base cohort had hypertension, and approximately half of the patients were prescribed corticosteroids and disease-modifying antirheumatic drugs and immunosuppressive medications.

Studies using RWD provide a unique opportunity to identify and observe large cohorts of patients over time, which is particularly useful for generating insights into rare or uncommon diseases. Nevertheless, limited RWD on the clinical characteristics and management of pediatric patients with CKD are currently available. In a multi‑institutional US-based EHR study, Gluck et al. identified 11,240 children with CKD out of over 7 million children across six pediatric health care organizations based on eGFR thresholds only (consisting of two eGFR values between 15 and < 90 mL/min/1.73 m^2^, measured at least 90 days apart; no UPCR criteria included). At the time of cohort entry, median age was 11 years, 50% of the cohort was female, 35% had comorbid hypertension, and 12% had CKD of glomerular etiology [26]. Findings were generally similar to those observed in our study, except for the proportion of patients who had CKD with glomerular etiology, which was substantially higher in our pediatric CKD cohort. Interestingly, only 37% of the overall cohort in the Gluck et al. study had proteinuria (which was included as an entry criterion for our study); nevertheless, in the sub-cohort with glomerular disease etiology, the proportion of patients with proteinuria was substantially higher (81%) [26].

The prospective Chronic Kidney Disease in Children (CKiD) study enrolled 586 children with eGFR between 30 and 90 mL/min/1.73 m^2^. In this population, the baseline median age was 11 years, 38% were female, and 54% had either hypertension at baseline or a history of hypertension with antihypertensive medication use [27]. Relative to the CKiD study, the cohort described here had a slightly older median age (14 years), a more balanced sex distribution (48% female), and fewer patients with hypertension at baseline (39%). Notably, glomerular etiology was apparent in 22% of the CKiD cohort [27], which was considerably lower than the proportion reported here. While proteinuria was not an inclusion criterion for the CKiD cohort, analysis of baseline data showed that 76% of patients had significant proteinuria (UPCR 0.2–2.0 g/g), and that proteinuria increased with decreasing GFR (as measured by iohexol disappearance) [27, 28]. It is worth noting that the threshold for the characterization of significant proteinuria in the CKiD study (UPCR 0.2–2 g/g) was lower than the levels used to define severely increased proteinuria in the current study, which were informed by criteria from the FIONA trial (≥ 0.5 g/g or ≥ 1 g/g, depending on age and CKD stage; Table 1) [28].

Previous reports on the epidemiology of pediatric CKD have indicated that congenital anomalies of the kidney and urinary tract (CAKUT) are the leading causes of CKD in children [9, 16, 26, 28]. CAKUT cases tend to be associated with a substantially lower degree of proteinuria than glomerular CKD [16, 28]. In the CKiD study, UPCR values were 140% higher in children with glomerular versus non-glomerular CKD etiology irrespective of eGFR [27, 28]. The fact that patients were required to have severely increased proteinuria for inclusion in the base cohort of the current study, as in FIONA, may at least partly explain why the majority of patients identified had CKD with a glomerular etiology, in contrast to these other studies.

Likewise, data from the 2024 US Renal Data System (USRDS) report on “Kidney Disease among Children and Adolescents” indicated that, among 38,230 children with CKD who were enrolled in Medicaid in 2022, structural disorders (a subset of CAKUT) were the most common cause of CKD across all ages analyzed. However, the proportion of children with this etiology decreased with increasing age (67% in children under 5 years old and 40% in those between the ages of 13 and 17 years). The opposite trend was observed for glomerular disorders as the cause of CKD [2]. Additionally, in a study that used EHR-derived algorithms to assess pediatric CKD incidence between 2014 and 2023 in the Atlanta Metropolitan Statistical Area, among 6814 pediatric patients with CKD, the highest incidence was reported among infants (< 1 year of age) followed by young children (1‒4 years of age), with CAKUT observed to be the most common etiology leading to CKD in young children [29]. Once again, most children in our study were older (≥ 6 years of age), and this difference is likely due to the selection criteria of patients with severely increased proteinuria in the FIONA trial, characteristic of children with glomerular disease, who tend to be older. The key differences noted between the findings of this study and those of previous RWD studies are that the FIONA trial population draws from a unique subset of the overall population of children with CKD—one that has not previously been extensively characterized or included in similar clinical investigations.

The FIONA trial aims to investigate the efficacy and safety of finerenone in pediatric patients with CKD and severely increased proteinuria. In a post hoc analysis of the FIDELITY dataset (pooled data from the FIDELIO-DKD and FIGARO-DKD trials), early finerenone-induced reductions in albuminuria (between baseline and 4 months after treatment initiation) were shown to mediate > 80% and > 30% of the beneficial effects of finerenone on kidney and CV outcomes, respectively, in adult patients with albuminuric CKD and type 2 diabetes [30]. In adult CKD, albuminuria is traditionally used as a predictor of CKD progression, whereas proteinuria has been favored in pediatric CKD studies because children more frequently have tubular CKD pathology than adults, which may be missed when testing for albuminuria only [4, 31]. However, it is also well recognized that proteinuria values may vary across patients, measurement periods, and studies according to the evaluation methods applied; these may include urinary dipstick reagent, sulfosalicylic acid turbidity tests, random or first morning urine for spot UPCR, and 24-h urinary protein and creatinine [32]. As a result of variations such as these, processes for collecting and reporting clinical trial data are highly controlled and standardized. In contrast, although RWD can offer broad external validity given use of data from routine clinical practice across geographies and health care settings, real-world lab measurements are not collected under the same standardized conditions in which they are collected in clinical trials. Therefore, the heterogeneity of recorded lab measures present in RWD must be considered to ensure reasonable and consistent assessments of outcomes derived from these routine measurements. In our study, UPCR was used to identify patients given its role as an eligibility criterion in the FIONA trial, and its relevance to the trial’s primary endpoint (change in UPCR over time) [19]. Data cleaning strategies were applied to mitigate any lack of comparability based on measurement setting/method. Nevertheless, certain conditions of the FIONA trial could not be applied to a real-world setting, which may have influenced the observed values in the current study. For example, in the FIONA trial, proteinuria is measured using the systematic collection of first morning voided urine samples over 3 consecutive days to derive an average UPCR. In Optum® EHR, the time of day when UPCR was measured is not available. This impacts our ability to understand the variability of real-world conditions at play that may impact observed UPCR values. As a result, noisiness in UPCR trajectories resulting from non-standard measurement conditions may be observed, posing a challenge for future longitudinal studies utilizing similar data.

Finally, the real-world use of ACEi/ARB treatment in pediatric CKD warrants additional consideration. While ACEi/ARB treatment is commonly recommended in adults to attenuate CKD progression by regulating blood pressure and proteinuria, there is less clarity and limited evidence as to their optimal use in children [16]. Their use in children is recommended to maintain normal blood pressure, decrease proteinuria, and slow kidney disease progression [15, 33]. In this study, all patients were required to have received a baseline ACEi/ARB prescription in alignment with dose optimization protocols implemented during the run-in phase of the FIONA trial [19]. Baseline sociodemographic and clinical characteristics were generally similar between the sensitivity and base cohorts, except for hypertension prevalence, which was substantially lower in the sensitivity cohort (16% vs. 39% in the base cohort). This suggests that pediatric patients with CKD in real-world clinical practice may only receive ACEi/ARB treatment if they present with high blood pressure.

Our findings indicate that the timing of ACEi/ARB initiation is a particularly important consideration in pediatric patients to ensure the robustness of future analyses using RWD in combination with trial data. The ACEi/ARB use eligibility criterion in this study had a substantial impact on the final sample size of the base cohort, resulting in the exclusion of 51% of patients in the final step of patient selection. A post hoc sensitivity analysis found that over 20% of the treatment-naïve patients who were excluded in the final step subsequently initiated ACEi/ARBs within the first 6 months of follow-up. This suggests that treatment-naïve patients did not remain untreated throughout their disease course and would likely have been eligible for inclusion in the base cohort at a later time point. These findings should be considered in the design of future studies using EHR data aimed at exploring disease progression in real-world pediatric patients with CKD. Beyond this, future studies should also consider the relevance of treatment stabilization periods included in clinical trials. The FIONA trial has an up to 90-day run-in period consisting of 60 days of ACEi/ARB initiation and/or optimization, followed by at least 30 days of stabilization [19]. ACEi/ARB initiation has been known to impact proteinuria; therefore, investigators need to ensure that changes in UPCR and eGFR correspond with disease progression rather than initial responses to ACEi/ARB commencement [16].

A strength of this study is its use of a large EHR dataset, which allows for the generation of RWD on a relatively small pediatric population that is largely underrepresented in the literature. Nevertheless, there are important limitations associated with using RWD derived from EHR. Data used in our study were generated from assessments made during routine clinical practice. However, unlike in a clinical trial environment, laboratory tests and other procedures are not performed at predefined intervals; thus, non-random missingness of data on key clinical markers may be observed in the real world. Notably, certain data limitations and missingness may be more pronounced in healthier patients, owing to fewer health care professional interactions and contributing to fewer data points. The lack of per protocol treatment in the real world compared with the controlled environment of a clinical trial can, in turn, make it more difficult to reduce the impact of other treatments as confounding factors. In some cases, patients may have received care from outside the provider network included in Optum® EHR, resulting in the possible incompleteness of data, which cannot be effectively assessed. Finally, given differences in the treatment and health care landscape, the findings of this study may not be generalizable to other countries outside of the United States. Studies similar to ours conducted in other countries may prove to be equally informative.

In conclusion, our study demonstrated the feasibility as well as the challenges associated with the use of EHR data to identify and describe pediatric patients with CKD and severely increased proteinuria based on adapted criteria from the FIONA trial. Our findings highlight important considerations with regard to the design of future studies exploring disease progression among pediatric patients with CKD with severely increased proteinuria in a real-world setting.

## Supplementary Information

Below is the link to the electronic supplementary material.

ESM 1 Graphical abstract (PPTX 107 KB)

ESM 2 (DOCX 280 KB)

ESM 3 (DOCX 44 KB)

## Data Availability

Data used for this research are commercially available in a deidentified format and can be accessed through a licensing agreement with Optum. Terms of agreement are specific to each client.
