# Supplementary material for: Characterizing children with chronic kidney disease and severe proteinuria in the United States: a retrospective analysis using electronic health records
Source: Pediatr Nephrol. 2026 Jul 6;41(12):4487–500. doi: 10.1007/s00467-026-07435-y (PMC13672603; doi:10.1007/s00467-026-07435-y)
Supplement: Supplementary file 1 [file 467_2026_7435_MOESM2_ESM.docx]

Supplementary Information

**Characterizing children with chronic kidney disease and severe proteinuria in the United States: a retrospective analysis using electronic health records**

*Pediatric Nephrology*

Bradley A. Warady,^1^ Simone Heeg,^2^ Alfredo E. Farjat,^3^ Rachel Knapp,^4^ Siir Su Saydam,^5^* Alexander Vowinkel,^2^ Alain Gay,^6^* James Potts,^7^ Andrea Scalise,^8^ Gordana Atanackovic,^9^ Sascha van Boemmel-Wegmann^5^*

^1^Department of Pediatrics, Division of Pediatric Nephrology, University of Missouri-Kansas City School of Medicine, Children’s Mercy Kansas City, Missouri, USA; ^2^Real World Data Science & AI (RWDS&AI), Global Medical & Evidence (GME), Bayer AG, Berlin, Germany ^3^Bayer BV, Clinical Statistics & Analytics, Hoofddorp, NL; ^4^Real World Evidence Center of Excellence (RWE CoE), Global Medical & Evidence (GME), Bayer AG, Berlin, Germany; ^5^Medical Affairs & Pharmacovigilance (MAPV), Integrated Evidence Generation (IEG), Bayer AG, Berlin, Germany; ^6^Studies and Pipeline, Global Medical Affairs (GMA), Bayer AG, Berlin, Germany; ^7^Bayer HealthCare Pharmaceuticals Inc., Whippany, NJ, USA; ^8^Cardiology and Nephrology Clinical Development, Bayer Hispania S.L., San Joan Despi, Spain; ^9^Research & Development, Pharmaceutical, Bayer US LLC, Whippany, NJ, USA

*Affiliation at the time of study conduct

**Corresponding author**

Name: Rachel Knapp

Address: Bayer AG, Müllerstr. 178, 13353 Berlin DE

Telephone number: +491735604348

Email address: [rachel.knapp@bayer.com](mailto:rachel.knapp@bayer.com)

**Supplementary Table 1 Baseline characteristic data collected**

| **Characteristic** | **Measures, operational definitions and categories** |
| --- | --- |
| ***Sociodemographic*** | |
| Age | Age (continuous) |
|  | Age group; categories: ≥6 months to <2 years; ≥2 to <6 years; ≥6 to <12 years; ≥12 to <18 years |
|  | Gestational age at birth or LBW/ELBW* |
| Sex | Categories: male, female |
| Race | Categories: White, Black or African American, Asian, other/unknown |
| Height | Continuous |
| ***Clinical*** | |
| CKD etiology | Categories: glomerular disease, non-glomerular disease, unknown* |
| CKD stage | CKD stages (CKiD U25) |
| Comorbidities | Presence of comorbidities defined by ≥1 diagnosis or procedure code in patient history* |
| Lab values | eGFR (continuous, categorical: ≥30 to <60, ≥60 mL/min/1.73 m^2^) |
|  | UPCR (continuous, categorical: ≥0.5–≤1.0, >1.0 g/g) |
|  | Serum potassium (continuous, categorical: ≤4.5, >4.5 mmol/L) |
|  | Serum albumin (continuous) |
|  | UACR (continuous) |
|  | Serum creatinine (continuous) |
| Blood pressure | SBP (continuous) |
|  | Diastolic blood pressure (continuous) |
| ***Comedications*** | |
| Antiarrhythmics | Prescription of antiarrhythmic medications (alpha- and beta-blocking drugs, selective beta-blocking drugs, non-selective beta-blocking drugs, calcium channel blockers with antiarrhythmic effects) |
| Diuretics | Prescription of diuretic medications (low-ceiling diuretics excluding thiazides, low-ceiling diuretics [thiazides]; diuretic carbonic anhydrase inhibitors; loop or high-ceiling diuretics; vasopressin antagonists; osmotic diuretics; other diuretics; potassium-sparing diuretics [epithelial sodium channel blocker diuretics, MRA]) |
| Antihypertensives | Prescription of antihypertensive medications (dihydropyridine derivative calcium channel blockers, other antihypertensive calcium channel blockers, adrenergic receptor agonists, ACE inhibitors, angiotensin receptor antagonists, renin inhibitors, other antihypertensives) |
| Others | Prescription of other medications (potassium supplements; SGLT2 inhibitors; erythropoiesis-stimulating drugs; corticosteroids; antineoplastics; immunosuppressants; CYP3A inhibitors; CYP3A inducers; strong CYP3A4 inhibitors; strong CYP3A4 inducers; antibacterials; immunomodulators; disease-modifying antirheumatic drugs; sodium chloride supplements; sacubitril- or sacubitrilat-containing drugs (ARNi); alkali agents; potassium binders) |

*Please see Supplementary Table 2 for lists of associated codes. ACE, angiotensin-converting enzyme; ARNi, angiotensin receptor–neprilysin inhibitor; BMI, body mass index; CKD, chronic kidney disease; CKiD, Chronic Kidney Disease in Children; CPT, Current Procedural Terminology; CYP3A, cytochrome P450-3A isozyme; CYP3A4, cytochrome P450 3A4; eGFR, estimated glomerular filtration rate; EHR, electronic health record; ELBW, extremely low birth weight; HCPCS, Healthcare Common Procedure Coding System; ICD-CM, International Classification of Diseases, Clinical Modification; LBW, low birth weight; MRA, mineralocorticoid receptor antagonist; NDC, National Drug Code; OLE, open-label extension; PCS, Procedure Coding System; SBP, systolic blood pressure; SGLT2, sodium-glucose cotransporter 2; STORK-CKD, Real-world evidence STudy using US lOngitudinal patient-level data to support Randomized clinical trial in pediatric Chronic Kidney Disease population; UACR, urine albumin-to-creatinine ratio; UPCR, urine protein-to-creatinine ratio.

Supplementary Table 2. Diagnostic and procedure codes used to define medical concepts

| **Medical concept** | **Ontology** | **Codes** |
| --- | --- | --- |
| *CKD etiology* | | |
| Glomerular disease | ICD-9-CM | 250.4, 287.0, 403.00–403.91, 405.01, 405.11, 405.91, 446.0, 446.21, 446.4, 580, 581, 582, 582.1–582.9, 583.6, 583.7, 701.0, 709.1, 710.1, 753.3 |
|  | ICD-10-CM | A36.84, B26.83, B58.83, D69.0, D86.84, E10.2, E11.2, I12, I15.0, L94.0, L95.8, M30.2, M31.0, M31.31, M31.8, M32.14, M32.15, M34, N00, N00.0–N00.A, N01, N02.4, N02.6–N02.A, N03, N04, N04.0–N04.A, N05, N06.A, N07.0, N07.1–N07.5, N07.7, N07.A, N08, N11.0, N11.8, N11.9, N12, N14, N15.8, N15.9, N26.9, Q63.9, Q87.81 |
| Kidney biopsy | ICD-9-CM | 55.23, 55.24, 55.3, 55.32, 55.33, 55.35, 55.39, 59.21, 59.91 |
|  | ICD-10-PCS | 0TB00ZX, 0TB00ZZ, 0TB03ZX, 0TB03ZZ, 0TB04ZX, 0TB04ZZ, 0TB07ZX, 0TB07ZZ, 0TB08ZX, 0TB08ZZ, 0TB10ZX, 0TB10ZZ, 0TB13ZX, 0TB13ZZ, 0TB14ZX, 0TB14ZZ, 0TB17ZX, 0TB17ZZ, 0TB18ZX, 0TB18ZZ, 0TB30ZX, 0TB33ZX, 0TB34ZX, 0TB37ZX, 0TB38ZX, 0TB40ZX, 0TB43ZX, 0TB44ZX, 0TB47ZX, 0TB48ZX |
|  | CPT | 50200, 50205, 50280, 50555, 50557, 50574, 50576, 52007, 52354 |
| Non-glomerular disease | ICD-9-CM | 270.0, 271.8, 583.81, 590.00, 590.01, 590.80, 590.81, 591, 592.0, 592.9, 593.0, 593.3, 593.4, 593.5, 593.71–593.73, 593.9, 599.6, 599.60, 599.69, 741, 747.62, 753.0, 753.6, 753.12–753.17, 753.20, 759.5, 759.6 |
|  | ICD-10-CM | E72.04, E72.09, E72.11, E72.53, N11.1, N13, N13.0–N13.5, N13.7–N13.9, N16, N20.0, N28.83, N29, Q27.1, Q05, Q60, Q60.0–Q60.5, Q61.2–Q61.5, Q62.39, Q64.2, Q85.1, Q85.9 |
| *Comorbidities* | | |
| Diabetes Mellitus | ICD-9-CM | 250 |
|  | ICD-10-CM | E10, E11 |
| Congenital heart malformation | ICD-9-CM | 745, 745.0–745.3, 745.6–745.8, 746, 746.0–746.9 |
|  | ICD-10-CM | Q20–Q24 |
| Heart failure | ICD-9-CM | 398.91, 402.01, 402.11, 402.91, 404.01, 404.03, 404.11, 404.13, 404.91, 404.93, 428 |
|  | ICD-10-CM | I09.81, I11.0, I13.0, I13.2, I50 |
| Cardiomyopathy | ICD-9-CM | 425 |
|  | ICD-10-CM | I42 |
| Urinary tract infection | ICD-9-CM | 590.1, 590.2, 590.8, 595.0, 595.9, 597.80, 599.0, 771.82 |
|  | ICD-10-CM | N10, N12, N15.1, N30.0, N30.9, N34.1, N34.2, N39.0 |
| Hypertension | ICD-9-CM | 362.11, 401, 402, 403, 404, 405, 437.2 |
|  | ICD-10-CM | H35.03, I10–I16, I67.4 |
| Growth retardation | ICD-9-CM | 779.34, 783.4 |
|  | ICD-10-CM | P92.6, R62.5 |
| Anemia | ICD-9-CM | 280–282284, 285 |
|  | ICD-10-CM | D50–D56, D57, D57.0, D57.00– D57.03, D57.09, D57.1, D57.2, D57.20, D57.21, D57.211–D57.213, D57.218, D57.219, D57.3, D57.4, D57.40, D57.41, D57.411–D57.413, D57.418, D57.419, D57.42, D57.43, D57.431–D57.433, D57.438, D57.439, D57.44, D57.45, D57.451–D57.453, D57.458, D57.459, D57.8, D57.80, D57.81, D57.811–D57.813, D57.818, D57.819, D58–D60, D61, D61.0, D61.01, D61.09, D61.1–D61.9, D62–D64 |
| Hyperlipidemia | ICD-9-CM | 272.0–272.4 |
|  | ICD-10-CM | E78.0–E78.5 |
| Metabolic bone disease | ICD-9-CM | 275.8, 275.9, 588.0 |
|  | ICD-10-CM | E83.89, E83.9, N25.0 |
| Prematurity | ICD-9-CM | 765.01–765.03, 765.1, 765.21–765.28 |
|  | ICD-10-CM | P07.0–P07.3 |
| *Exclusion criteria* | | |
| Prior kidney transplant | ICD-9-CM | 55.6, 996.81, V42.0 |
|  | ICD-10-CM | T86.1, Z94.0 |
|  | ICD-10-PCS | 0TY00Z0–0TY00Z2, 0TY10Z0–0TY10Z2 |
|  | CPT | 00868, 50300, 50320, 50323, 50325, 50327, 50328, 50329, 50360, 50365, 50370, 50380 |
| Other urological surgery | ICD-9-CM | 55.39, 55.4, 55.51, 56.42, 57.59, 57.6, 57.79, 59.91, 60.29, 60.3, 60.4, 60.61, 60.69, 60.82 |
|  | ICD-10-PCS | 0TB00ZZ, 0TB04ZZ, 0TB10ZZ, 0TB14ZZ, 0TBB0ZZ, 0TBB4ZZ, 0TT00ZZ, 0TT04ZZ, 0TT10ZZ, 0TT14ZZ, 0TT60ZZ, 0TT64ZZ, 0TT70ZZ, 0TT74ZZ, 0TTB0ZZ, 0TTB4ZZ, 0VB00ZZ, 0VB04ZZ, 0VB07ZZ, 0VB08ZZ, 0VT00ZZ, 0VT04ZZ, 0VT07ZZ, 0VT08ZZ |
|  | CPT | 50220, 50225, 50230–50236, 50240, 50543, 50545, 50546, 50548, 51550, 51555, 51565, 51570, 51575, 51580, 51585, 51590, 51595, 51596 |
| HUS | ICD-9-CM | 283.11 |
|  | ICD-10-CM | D59.3 |
| Albumin infusion | HCPCS | P9041, P9045, P9046, P9047 |
| Renal artery stenosis | ICD-9CM | 440.1 |
|  | ICD-10-CM | 170.1, Q27.1 |
| Kidney failure | ICD-9-CM | 584, 585.5, 585.6, 586 |
|  | ICD-10-CM | N17, N18.5, N18.6, N19, N99.0 |
| Dialysis | ICD-9-CM | 38.95, 39.27, 39.42, 39.43, 39.95, 54.98, 458.21, 792.5, 996.56, 996.68, 996.73, E872.2, E879.1, V45.1, V56.0–V56.3, V56.31, V56.32, V56.8 |
|  | ICD-10-CM | I95.3, R88.0, T81.502, T81.512, T81.522, T81.532, T81.592, T82.4, T85.611, T85.621, T85.631, T85.691, T85.71, Y62.2, Y84.1, Z49.0, Z91.15, Z99.2 |
|  | CPT | 0505F, 0507F, 36800, 36810, 36815, 36831–36833, 36838, 36901–36903, 4052F–4055F, 49421, 75791, 90935, 90937, 90940, 90945, 90947, 90951–90958, 90966–90969, 90989, 90993, 90999, 93990, 99512 |
|  | HCPCS | A4653, A4663, A4671–A4674, A4680, A4690, A4706–A4709, A4714, A4719–A4726, A4728, A4730, A4740, A4750, A4755, A4760, A4765–A4766, A4770, A4774, A4802, A4860, A4870, A4890, A4913, A4918, A4929, C1750, C1752, C1881, E1510, E1520, E1530, E1540, E1550, E1560, E1575, E1580, E1590, E1592, E1594, E1600, E1610, E1615, E1620, E1625, E1630, E1632, E1634–E1637, E1639, E1699, G0257, G0491, G0492, J0882, J0886, J0887, Q0139, Q4081, S9335, S9339 |
| Pregnancy | ICD-9-CM | 630–679, V72.42 |
|  | ICD-10-CM | O68, O76, O82 |
|  | ICD-10-PCS | 0W8NXZZ, 10D00Z0–10D07Z8, 10D17Z9–10D18ZZ, 10E0XZZ |
|  | CPT | 59050, 59051, 59400, 59409, 59410, 59414, 59430, 59510, 59514, 59515, 59525, 59610, 59612, 59614, 59618, 59620, 59622, 76801, 76802, 99464, 99465 |
|  | HCPCS | G9356 |
| Dent’s disease | ICD-9-CM | 599.89 |
|  | ICD-10-CM | N39.8 |
| Addison’s disease | ICD-9-CM | 255.9, 255.41 |
|  | ICD-10-CM | E27.1, E27.2, E27.9 |
| Significant liver disease or liver failure | ICD-9-CM | 070.0, 070.2, 070.41, 070.43, 070.44, 070.49, 070.51, 070.53, 070.6, 070.71, 456.0, 456.1, 570, 571, 571.0–571.3, 571.41, 571.42, 571.49, 571.5–571.8, 572, 573.1, 573.2, 573.4, 573.5, 456.0, 456.1, V42.7 |
|  | ICD-10-CM | B15.0, B16, B17, B19, B19.0, B19.11, B19.21, K70, K70.0–K70.4, K71, K72, K73.2, K74, K74.02, K74.1–K74.6, K75.0, K75.1, K75.3–K75.9, K76.1–K76.7, K76.81 |
| Malignancy | ICD-9-CM | 140–209 |
|  | ICD-10-CM | C00–C96 |

In cases where all associated daughter codes were used, only the parent code is listed. CPT, Current Procedural Terminology; HCPCS, Healthcare Common Procedure Coding System; HUS, hemolytic-uremic syndrome; ICD-9-CM, International Classification of Diseases Clinical Modification, 9^th^ Revision; ICD‑10‑CM, International Classification of Diseases, 10^th^ Revision, Clinical Modification; ICD-10-PCS, International Classification of Diseases, 10^th^ Revision, Procedure Coding System.

Supplementary Table 3 Study population inclusion/exclusion criteria

| **FIONA/FIONA OLE** | **STORK-CKD** |
| --- | --- |
| ***Inclusion criteria*** | |
| 6 months to <18 years of age at the time informed consent/assent is signed | 6 months to <18 years of age on the index date as identified from patient demographic information |
| Clinical diagnosis of CKD at screening, defined as:   - CKD stages 1–3 (eGFR ≥30 mL/min/1.73 m^2^) for children ≥1 to <18 years of age or - An SCr ≤0.40 mg/dL for infants 6 months to <1 year of age and severely increased proteinuria as defined by UPCR:   - ≥0.50 g/g in participants ≥2 years of age with CKD stage 2 and 3 or   - ≥1.0 g/g for patients <2 or ≥2 years of age with CKD stage 1 | At least 2 pairs of lab values indicating CKD defined by eGFR/SCr value and severely increased proteinuria defined by UPCR (with up to 89 days between the 2 measurements) and between 90 and 365 days between the pairs:   - Children 2 to <18 years of age at measurement - eGFR 30 to <90 mL/min/1.73 m^2^ and UPCR ≥0.5 g/g or - eGFR value ≥90 mL/min/1.73 m^2^ and UPCR ≥1.0 g/g - Children 1 to <2 years of age at measurement - eGFR value ≥30 mL/min/1.73 m^2^ and UPCR ≥1.0 g/g - Children 6 months to <1 year of age at measurement - SCr ≤0.4 mg/dL and UPCR ≥1.0 g/g |
| N/A | At least 180 days of EHR activity prior to index date (defined as the baseline period) |
| Stable kidney function between screening and day 0, defined as^a^:   - No increase or decrease in eGFR ≥15% for children ≥1 year of age - No increase or decrease in creatinine ≥0.10 mg/dL for children <1 year of age | *See kidney function exclusion criteria below* |
| Treated with an ACEi/ARB at optimized doses defined as maximally tolerable doses within the recommended dose range according to guidelines on blood pressure management, unchanged for ≥30 days prior to screening | Record of ACEi/ARB use at baseline |
| Serum potassium of ≤5.0 mmol/L for children ≥2 years of age at both screening and day 0, and ≤5.3 mmol/L for children <2 years of age | *See serum potassium exclusion criteria below* |
| Enrolment allowed in outpatient or inpatient settings | All children with a record in Optum^®^ EHR included, regardless of treatment setting |
| Body weight ≥4.0 kg at visit 1 | Body-weight information not used (measurements inconsistent in Optum^®^ EHR due to missing data and considered non-essential for real-world translation for the population of interest) |
| ***Exclusion criteria*** | |
| *See CKD inclusion criteria above* | Severe CKD   - Children 1 to <18 years of age at measurement   - At least 2 values of eGFR <30 mL/min/1.73 m^2^ in the baseline period ≥90 days apart - Children <1 year of age at measurement   - At least 2 values of SCr >0.4 mg/dL in the baseline period ≥90 days apart |
| Planned urological surgery expected to influence kidney function | - *Censoring criterion^b^* - *Prior kidney transplant* |
| *See kidney function inclusion criteria above* | Unstable kidney function based on of 2 eGFR or SCr measurements within 30 days prior to and including index date   - Children ≥1 year to 18 years of age at measurement   - An increase or decrease in eGFR ≥15% - Children 6 months to <1 year of age at measurement   - An increase or decrease in SCr ≥0.1 mg/dL   (If only 1 value of eGFR was available in this time frame, the patient was considered to have stable kidney function) |
| *See serum potassium inclusion criteria above* | Serum potassium based on the age at measurement   - >5.0 mmol/L for children ≥2 to 18 years of age at measurement - >5.3 mmol/L for children 6 months to <2 years of age at measurement   (Based on ≥2 predetermined serum potassium lab values on inpatient records 72 hours apart or ≥1 predetermined serum potassium lab value on the outpatient record with one such value on the inpatient or outpatient records no longer than 7 days apart within 30 days prior to index date) |
| HUS diagnosed ≤6 months prior to screening | At least 1 inpatient or 2 outpatient diagnosis codes for HUS in the baseline period |
| Nephrotic syndrome and receiving albumin infusions within the last 6 months prior to screening | Albumin infusion procedure or prescription code in any time in the baseline period |
| Scheduled for kidney transplantation within the study time frame | Procedure or diagnosis code for kidney transplant or allograft at any time prior to the index date |
| Kidney allograft in place |  |
| Bilateral renal artery stenosis | At least 1 inpatient or 2 outpatient diagnosis codes for bilateral renal artery stenosis at any time prior to index date |
| AKI requiring dialysis within 6 months prior to screening | At least 1 inpatient or 2 outpatient records with a kidney failure diagnosis code within the baseline period |
|  | At least 1 dialysis procedure or diagnosis code^c^ within the baseline period |
| Genetically defined primary tubulopathies leading to tubular proteinuria, such as Dent’s disease | At least 1 inpatient or 2 outpatient diagnosis codes for Dent’s disease within the baseline period |
| Systemic hypertension stage 2 in children ≥1 year of age defined according to guidelines on blood pressure management at screening or randomization | Fluctuating BP defined by a calculated MAD cutoff of 10 mmHg across all SBP values within the 180 days prior to index. |
|  | Median SBP value indicating systemic hypertension stage 2 in children ≥1 to 18 years of age at measurement (across all available values within 90 days prior to index date and defined according to guidelines on BP management) |
| SBP >110 mmHg in infants 6 months to <1 year of age at screening or randomization | Median SBP >110 mmHg in children 6 months to <1 year of age at measurement (across all available values within 90 days prior to index date) |
| Systemic hypotension defined as SBP below the 5th percentile for age, sex, and height at either screening or randomization, but no lower than 80 mmHg  (Exclusion if the 5th percentile of SBP is <80 mmHg) | Median SBP corresponding to systemic hypotension below the 5th percentile for age, sex, and height within 90 days prior to index date. (Children were also excluded if 5th percentile of SBP is <80 mmHg and median SBP is <80 mmHg) |
| Known hypersensitivity to the study treatment (active substance or excipients) | N/A |
| Addison’s disease | At least 1 inpatient or 2 outpatient diagnosis codes for Addison’s disease at any time prior to index date |
| Severe hepatic insufficiency defined by Child-Pugh C or analogous scores | At least 1 inpatient or 2 outpatient diagnosis codes for significant liver disease or liver failure within the baseline period |
| Use of rituximab, cyclophosphamide, abatacept, or intravenous glucocorticoids for any reason within <6 months prior to screening | Prescription code for rituximab, cyclophosphamide, abatacept, or intravenous glucocorticoids within the baseline period |
| Concomitant therapy with an MRA (eplerenone, spironolactone, esaxerenone, canrenone), any renin inhibitor (aliskiren, enalkiren, remikiren), any SGLT2i, sacubitril/valsartan combination (ARNi), or potassium-sparing diuretic (amiloride, triamterene) within 30 days prior to the screening visit | *See Supplementary Table 2* |
| Concomitant therapy with both ACEi and ARBs within 30 days prior to the screening visit | *See Supplementary Table 2* |
| Concomitant therapy with strong CYP3A4 inhibitors, or moderate or strong CYP3A4 inducers within 7 days prior to randomization | *See Supplementary Table 2* |
| Previous assignment to treatment during this study | N/A |
| Simultaneous participation in another interventional clinical study (e.g., phase 1–4 clinical studies) or treatment with any investigational medicinal product within 30 days prior to run-in visit | N/A |
| Any other condition or therapy, which would make the participant unsuitable for this study and not allow participation for the full planned study period (e.g., active malignancy or other condition limiting life expectancy to less than 6 months) | At least 1 inpatient or 2 outpatient diagnosis codes for malignancy in the baseline period |
| Any other history, condition, therapy, or uncontrolled intercurrent illness which could in the opinion of the investigator affect compliance with study requirement | N/A |
| Pregnant, breastfeeding, or intention to become pregnant during the study | *Censoring criterion^b^* |

^a^In the period since the current analysis was designed, the definition for the “stable kidney function between screening and day 0” inclusion criterion has been updated in the FIONA trial protocol. Updated criterion definition: for participants with a creatinine level of >0.8 mg/dL at screening, no increase or decrease in eGFR by ≥20% at day 0; for participants with a creatinine level of ≤0.8 mg/dL at screening, no increase or decrease in creatinine ≥0.15 mg/dL at day 0. ^b^Censoring criteria included urological surgery, pregnancy, end-of-data availability or death, whichever came first. ^c^For the application of this exclusion criterion in real-world data, kidney failure diagnosis and dialysis criteria were specified separately instead of AKI requiring dialysis because children on dialysis were excluded from the RCT as a standalone criterion, which is reflected in the exclusion criteria listed above, and children with kidney failure were not eligible for the trial by default.

ACEi, angiotensin-converting enzyme inhibitor; AKI, acute kidney injury; ARB, angiotensin receptor blocker; ARNi, angiotensin receptor–neprilysin inhibitor; BP, blood pressure; CKD, chronic kidney disease; CYP3A4, cytochrome P450 3A4; eGFR, estimated glomerular filtration rate; EHR, electronic health record; HUS, hemolytic uremic syndrome; MAD, median absolute deviation; MRA, mineralocorticoid receptor antagonist; N/A, not applicable; OLE, open-label extension; RCT, randomized controlled trial; SBP, systolic blood pressure; SCr, serum creatinine; SGLT-2i, sodium-glucose cotransporter 2 inhibitor; STORK-CKD, Real-world evidence STudy using US lOngitudinal patient-level data to support Randomized clinical trial in pediatric Chronic Kidney Disease population; UPCR, urine protein-to-creatinine ratio.

Supplementary Fig. 1 STORK-CKD study design

^a^Assessment windows varied per covariate. ^b^Exclusion criteria assessment 1: Not having stable kidney function, hyperkalemia (K^+^ >5.0 mmol/L for children ≥2 years of age or >5.3 mmol/L for <2 years of age). ^c^Exclusion criteria assessment 2: Having systemic hypertension stage 2 as defined by guidelines, SBP >110 mmHg in infants 6 months to <1 year of age, systemic hypotension defined as a SBP below the 5th percentile. ^d^Exclusion criteria assessment 3: Severe CKD (eGFR <30 mL/min/1.73 m^2^ for children ≥1 year of age or SCr >0.4 mg/dL for 6 months to <1 year), HUS, albumin infusion, kidney failure, dialysis, significant liver disease/liver failure, prescriptions (rituximab, cyclophosphamide, abatacept, or intravenous glucocorticoids), active malignancy. ^e^Exclusion criteria assessment 4: Kidney transplant or allograft procedure, bilateral renal artery stenosis, Addison’s disease. ^f^Censoring criteria: urological surgery, end-of-data availability, death, pregnancy.

CKD, chronic kidney disease; eGFR, estimated glomerular filtration rate; EHR, electronic health record; HUS, hemolytic uremic syndrome; SBP, systolic blood pressure; SCr, serum creatinine; STORK-CKD, Real-world evidence STudy using US lOngitudinal patient-level data to support Randomized clinical trial in pediatric Chronic Kidney Disease population.

**Supplementary Fig. 2 Patient identification in Optum^®^ EHR**

^a^Two laboratory values each for UPCR and eGFR, taken 90–365 days apart per pair of measurements

ACEi, angiotensin-converting enzyme inhibitor; ARB, angiotensin receptor blocker; BP, blood pressure; CKD, chronic kidney disease; eGFR, estimated glomerular filtration rate; EHR, electronic health record; HUS, hemolytic uremic syndrome; IV, intravenous; UPCR, urine protein-to-creatinine ratio.
